# A ϱ-Weyl fractional operator of the extended S-type function in a complex domain

**DOI:** 10.1016/j.mex.2024.103061

**Published:** 2024-11-17

**Authors:** Sarem H. Hadi, Khalid A. Challab, Ali Hasan Ali, Abdullah A. Alatawi

**Affiliations:** a Department of Mathematics, College of Education for Pure Sciences, University of Basrah, Basrah 61001, Iraq; b Department of Business Management, Al-imam University College, Balad 34011, Iraq; c The General Directorate of Education at Diwaniyah, AlQadisiyah, Iraq; d Technical Engineering College, Al-Ayen University, 64001 Dhi Qar, Iraq; e Department of Mathematical science, King Abdullah Air Defence Academy, 21944 Taif, Mecca, Saudi Arabia

**Keywords:** The S-Type Function, The s-function, Fractional integral operators, Fractional differential operators, ϱ-weyl fractional operator

## Abstract

This paper proposes a new extension of the well-known S-special function, which is called SM-function. We introduce this function by drawing inspiration from exponential function. This new special function is studied from a variety of analytical perspectives, including differential and integral operators. Furthermore, the ϱ-Weyl fractional integral operator involving the SM-function is studied. These classes are defined by utilizing a new q-differential operator.

• The SM- function is provided.
• The derivative and integral formulas of the SM-function are studied.
• An application of the ϱ-Weyl fractional integral operator associated with the SM-function is investigated.

## Graphical abstract

**Figure:**
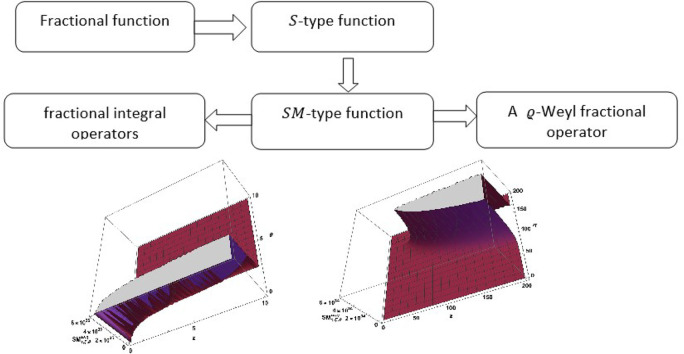

Specifications table

**Table:** 

Subject area:	Mathematics
More specific subject area:	Fractal-Fractional calculus
Name of your method:	The S-Type Function
Name and reference of original method:	The name of the method is: S-Type Function Reference 1. H. Amsalu, D. L. Suthar, Generalized fractional integral operators involving Mittag-Leffler function, abstract and Applied Analysis 8 (2018), pages Art. ID 7,034,124. Link: https://econpapers.repec.org/article/hinjnlaaa/7034124.htm 2. J. Saxena and R. K. Daiya, Integral transforms of the S-functions, Le Math. 700 (2015), 147–159. Link: https://lematematiche.dmi.unict.it/index.php/lematematiche/article/view/1167
Resource availability	N/A

## Method details

### Concepts

One important area of study in complex analysis is the theory of special functions, sometimes known as special series. Special functions are mathematical functions with specific consequences that are typically specified using integral representations, as solutions to several differential equations, or in the formula of a power series. The theory of special functions is strongly intertwined with the theory of Lie groups and Lie algebras, as well as some aspects of mathematical physics, due to the fundamental role that symmetries of differential equations play in both physics and mathematics. In 2015, Saxena and Daiya [29] provided and investigated a new function called S-function, its relation with other functions, which is a generalization of k-Mittag-Leffler function (MLF), k-function, Generalized M-series, M-series, generalized k-hypergeometric function, k-hypergeometric function, Mittag-Leffler function, and other several functions. All of these functions have been used to solve many problems in physical, mathematical, and engineering applications. In recent times, the exploration of the theory of special functions has garnered significant interest among researchers, owing to the imperative need to address problems that arise in several fields of knowledge. Special functions play a crucial part in the study of many special functions, since they are fundamental in the extensions and generalisations of these functions (e.g. see [7,9,12,17,18,35]). The S-function is specified for η,ϑ,ς,σ∈C,R(ϑ)>0,R(ϑ)>ϱR(σ),σ∈(0,1)∪N and ϱ∈R as

(1) $$S_{\tau ,\varsigma ,\vartheta }^{\varpi ,\eta }\left(j,\sigma ,\varrho ;z\right)=\sum_{j=0}^{\infty }\;\;\frac{\prod_{n=1}^{l}\;{\left(\varpi_{n}\right)}_{j}{\left(\eta \right)}_{j\sigma ,\varrho }}{\prod_{u=1}^{k}\;{\left(\tau_{u}\right)}_{j}\Gamma_{\varrho }\left(\vartheta j+\varsigma \right)}.\frac{z^{j}}{j!}.$$

In recent research, several writers have examined ϱ-fractional integral operators. To achieve this objective, we start by considering the subsequent characteristics documented in the literature. In 2007, Diaz and Pariguan [14] introduced the generalized ϱ-Gamma Function $\Gamma_{\varrho }\left(\eta \right)$ as

(2) $$\Gamma_{\varrho }\left(\eta \right)=\underset{n\to \infty }{lim}\frac{n!\varrho^{n}{\left(n\varrho \right)}^{\frac{\eta }{k}}-1}{{\left(\eta \right)}_{n,\varrho }},\;\left(\varrho >0,\eta \in C\setminus \varrho Z^{-}\right).$$

Here ${\left(\eta \right)}_{j,\varrho }$ is the ϱ-Pochhammer symbol given by:

(3) $${\left(\eta \right)}_{j,\varrho }=\left\{ \begin{array}{c} \frac{\Gamma_{\varrho }\left(\eta +j\varrho \right)}{\Gamma_{\varrho }\left(\eta \right)},\;\varrho \in R,\eta \in C/\left\{0\right\}\\ \eta \left(\eta +\varrho \right)\left(\eta +2\varrho \right)\cdots \left(\eta +\left(j-1\right)\varrho \right),\left(j\in N,\eta \in C\right), \end{array}\right.$$

where ϱ-Gamma function (see [22]) is also given by

(4) $$\Gamma_{\varrho }\left(z\right)=\int_{0}^{\infty }\;\;u^{z-1}e^{-\frac{u^{\varrho }}{\varrho }}du=\varrho^{\frac{z}{\varrho }-1}\Gamma \left(\frac{z}{\varrho }\right),\;\left(z\in C,\varrho \in R,R\left(z\right)>0\right).$$

The ϱ-Beta function (see [22]) is defined as

(5) $$B_{\varrho }\left(f,g\right)=\frac{1}{\varrho }\int_{0}^{1}\;\;u^{\frac{f}{\varrho }-1}{\left(1-u\right)}^{\frac{g}{\varrho }-1}du,\;\left\{R\left(f\right),\;R\left(g\right)\right\}>0.$$

The relation between ϱ-beta and ϱ-gamma functions can be given as

(6) $$B_{\varrho }\left(f,g\right)=\frac{\Gamma_{\varrho }\left(f\right)\Gamma_{\varrho }\left(g\right)}{\Gamma_{\varrho }\left(f+g\right)},\;\left(R\left(f\right),R\left(g\right)\right)>0.$$

Furthermore, provided other properties such as (also see [22])

(7) $$\Gamma_{\varrho }\left(z+\varrho \right)=\varrho \Gamma_{\varrho }\left(z\right),$$

(8) $${\left(\kappa \right)}_{n,\varrho }=\varrho^{n}{\left(\frac{\kappa }{\varrho }\right)}_{n},$$

(9) $${\left(\eta \right)}_{j\sigma ,\varrho }={\left(\varrho \right)}^{j\sigma }{\left(\frac{\eta }{\varrho }\right)}_{j\sigma },\;\left(\eta \in C,\varrho ,\sigma \in R,\;R\left(\eta \right)>0\right),$$

(10) $${\left(z\right)}_{j,\varrho }=\frac{\Gamma_{\varrho }\left(z+j\varrho \right)}{\Gamma_{\varrho }\left(z\right)},$$

(11) $${\left(z\right)}_{j+n,\varrho }={\left(z\right)}_{n,\varrho }{\left(z+n\varrho \right)}_{j,\varrho }.$$

Definition 1 **[16]**

*The*M*-series is defined as*

$$M_{l,k}^{\vartheta }\left(z\right)=\sum_{j=0}^{\infty }\;\frac{{\left(\varpi_{1}\right)}_{j},\ldots ,{\left(\varpi_{l}\right)}_{j}}{{\left(\gamma_{1}\right)}_{j},\ldots ,{\left(\gamma_{k}\right)}_{j}}.\frac{z^{j}}{\Gamma \left(\vartheta j+1\right)},$$

with ϑ∈C, R(ϑ)>0 and ${\left(\varpi_{l}\right)}_{j},{\left(\gamma_{k}\right)}_{j}$ are Pochammer symbols. Obviously, the series converges for all z when l≤k.

Definition 2 **[16]**

*The generalized*M*-series is provided as*

$$M_{l,k}^{\vartheta ,\varsigma }\left(z\right)=\sum_{j=0}^{\infty }\;\frac{{\left(\varpi_{1}\right)}_{j},\ldots ,{\left(\varpi_{l}\right)}_{j}}{{\left(\gamma_{1}\right)}_{j},\ldots ,{\left(\gamma_{k}\right)}_{j}}.\frac{z^{j}}{\Gamma \left(\vartheta j+\varsigma \right)},$$

with ϑ,ς∈C, R(ϑ)>0 and ${\left(\varpi_{l}\right)}_{j},{\left(\gamma_{k}\right)}_{j}$ are Pochammer symbols.

## Motivation and research objective

The solution of differential equation with fractional order makes extensive use of the special functions. Fractional calculus has emerged as a valuable tool for modeling and analysis, playing a crucial role in several domains such as material science, physics, mechanics, power systems, economics, and control theory. Recently, there has been an increased emphasis on the development of applications involving fractional calculus. When developing integration and differentiation using the fractional calculus powers of real or complex numbers, such as integral and differential operators, the fractional calculus is crucial. The reader may see [[1], [2], [3], [4], [5],^7^,^8^,^13^,^19^,^20^,[24], [25],[33], [35],^36^] for advancements in fractional calculus that are more recent.

The structure of this paper is as follows: Section (2) addresses a new concept of the special function, namely SM-function. Further, some of differential and integral operators properties with the SM-function are derived in Section (3). Results for ϱ-Weyl fractional integral related to the SM-functions is also examined in Section (4).

This section start by defining the main concept SM-function with some specific cases as in the Definition 4 below.

Definition 3 **[6]**

*The generalized* k *-hypergeometric function is defined as*

(12) $${\;}_{\;l}F_{k,j,v}\left[\varpi_{n};\tau_{u};z\right]=\sum_{j=0}^{\infty }\;\;\frac{\prod_{n=1}^{l}\;{\left(\varpi_{n}\right)}_{j,v}}{\prod_{u=1}^{k}\;{\left(\tau_{u}\right)}_{j,v}}\frac{z^{j}}{j!}.$$

Definition 4 *For*η,ϑ,ς,σ∈C,min{R(ϑ),R(ς),R(η)}>0,R(ϑ)>ϱR(σ),σ∈(0,1)∪N*and*ϱ∈R,*we provide the*SM*-function as follows:*

(13) $$SM_{\tau ,\varsigma ,\vartheta }^{\varpi ,\eta }\left(j,\sigma ,v,\varrho ;z\right)=\sum_{j=0}^{\infty }\;\;\frac{\prod_{n=1}^{l}\;{\left(\varpi_{n}\right)}_{j,v}{\left(\eta \right)}_{j\sigma ,\varrho }}{\prod_{u=1}^{k}\;{\left(\tau_{u}\right)}_{j,v}\Gamma_{\varrho }\left(\vartheta j+\varsigma \right)}.\frac{z^{j}}{j!},$$

where ${\left(\eta \right)}_{j,\varrho }$ is defined as (3) and ${\left(\eta \right)}_{j\sigma }=\frac{\Gamma (\eta +j\sigma )}{\Gamma (\eta )}=\sigma^{j\sigma }\prod_{n=1}^{\sigma }\;\;{\left(\frac{\eta +n-1}{\sigma }\right)}_{j}.$

Remark 1 *Some types of special functions of the* SM *-function are listed here:*

1. When v=1, we get S-function (see [29,34]).
2. When v=ϱ=1, we find R-function [23].
3. When v=ϱ=σ=1, we get K-function [23].
4. When v=ϱ=σ=η=1, we get a generalized M-series [32].
5. When v=ϱ=σ=η=ς=1, we have M-series [30].
6. When σ=η=ς=ϑ=1, we get a generalized ϱ-hypergeometric function [6].
7. When n=u=0,we find a generalized ϱ-Mittag-Leffler function (see [26,31]).
8. When n=u=σ=1,n=u=0,ϖ=γ,andτ=1, we get ϱ-Mittag-Leffler function [15].

**Fig. 1:**
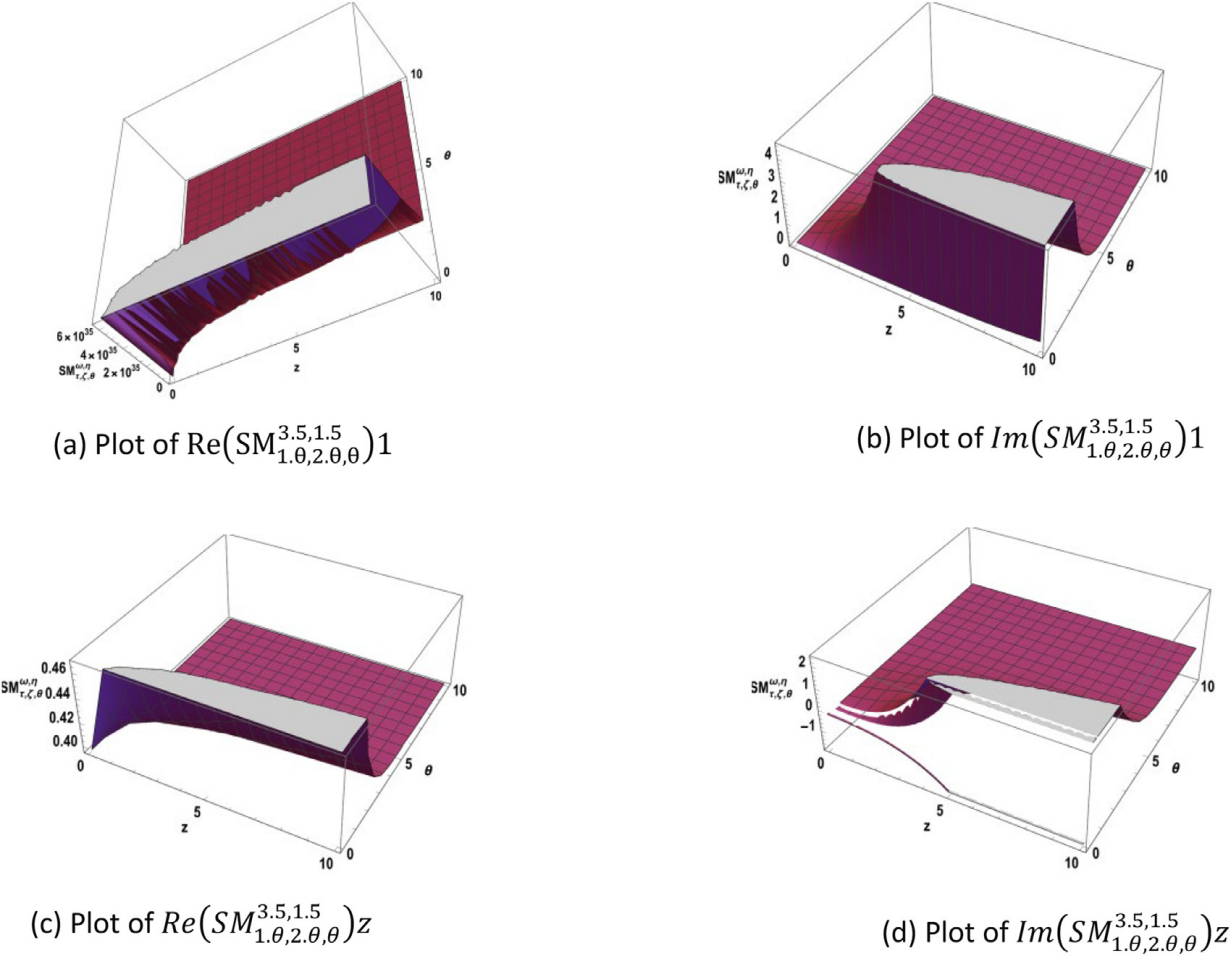
Represents the plots of the SM-function (13) in real and imaginary parts.

**Fig. 2:**
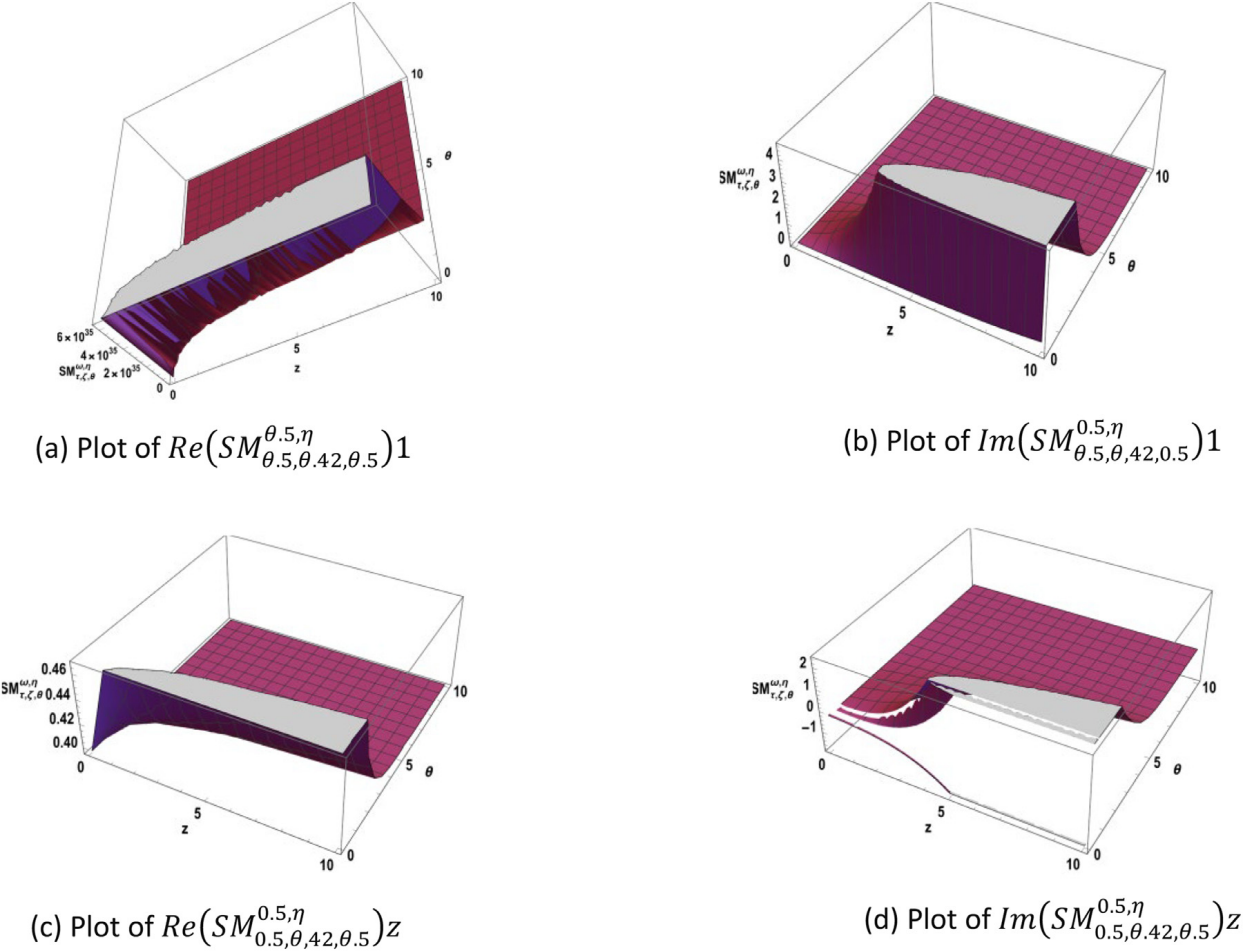
Represents another plots of the SM-function (13) in real and imaginary parts with different values.

The concept of the SM-function in (13) is illustrated in Figs. 1 and 2.

## Method validation

From Definition 4 and properties (3) and (4), we can get another form of the SM-function as the following

(14) $$\begin{array}{ccc} SM_{\tau ,\varsigma ,\vartheta }^{\varpi ,\eta }\left(j,\sigma ,v,\varrho ;z\right) & = & \varrho^{1-\frac{\varsigma }{\varrho }}\sum_{j=0}^{\infty }\;\;\frac{\prod_{n=1}^{l}\;{\left(\varpi_{n}\right)}_{j,v}{\left(\frac{\eta }{\varrho }\right)}_{j\sigma }}{\prod_{u=1}^{k}\;{\left(\tau_{u}\right)}_{j,v}\Gamma \left(\frac{\vartheta }{\varrho }j+\frac{\varsigma }{\varrho }\right)}.\frac{{\left(\varrho^{\sigma -\frac{\vartheta }{\varrho }}z\right)}^{j}}{j!}\\ & = & \varrho^{1-\frac{\varsigma }{\varrho }}SM_{\tau ,\frac{\varsigma }{\varrho },\frac{\vartheta }{\varrho }}^{\varpi ,\frac{\eta }{\varrho }}\left(j,\sigma ,v,\varrho ;\varrho^{\sigma -\frac{\vartheta }{\varrho }}z\right). \end{array}$$

Further,

(15) $$\varrho^{\frac{\varsigma }{\varrho }-1}SM_{\tau ,\frac{\varsigma }{\varrho },\frac{\vartheta }{\varrho }}^{\varpi ,\frac{\eta }{\varrho }}\left(j,\sigma ,v,\varrho ;\varrho^{\frac{\vartheta }{\varrho }-\sigma }pz\right)=SM_{\tau ,\frac{\varsigma }{\varrho },\frac{\vartheta }{\varrho }}^{\varpi ,\frac{\eta }{\varrho }}\left(j,\sigma ,v,\varrho ;pz\right),\;p\in R.$$

Theorem 1 *Let*η,σ,ϑ,ς∈C,min{R(ϑ)*,*R(ς),R(η)}>0*and*ϱ∈R,*then*

(16) $$SM_{\tau ,\varsigma ,\vartheta }^{\varpi ,\eta }\left(j,\sigma ,v,\varrho ;z\right)=\varsigma SM_{\tau ,\varsigma +\varrho ,\vartheta }^{\varpi ,\eta }\left(j,\sigma ,v,\varrho ;z\right)+\vartheta z{\left(SM_{\tau ,\varsigma +\varrho ,\vartheta }^{\varpi ,\eta }\left(j,\sigma ,v,\varrho ;z\right)\right)}^{\prime }$$

and we have

(17) $$\vartheta z{\left(SM_{\tau ,\varsigma +\varrho ,\vartheta }^{\varpi ,\eta }\left(j,\sigma ,v,\varrho ;z\right)\right)}^{\prime }=SM_{\tau ,\varsigma ,\vartheta }^{\varpi ,\eta }\left(j,\sigma ,v,\varrho ;z\right)-\varsigma SM_{\tau ,\varsigma +\varrho ,\vartheta }^{\varpi ,\eta }\left(j,\sigma ,v,\varrho ;z\right).$$

*Proof.* Starting on the left side of (16), we get

$$\varsigma SM_{\tau ,\varsigma +\varrho ,\vartheta }^{\varpi ,\eta }\left(j,\sigma ,v,\varrho ;z\right)+\vartheta z{\left(SM_{\tau ,\varsigma +\varrho ,\vartheta }^{\varpi ,\eta }\left(j,\sigma ,v,\varrho ;z\right)\right)}^{\prime }$$

$$\;=\sum_{j=0}^{\infty }\;\;\frac{\varsigma \prod_{n=1}^{l}\;{\left(\varpi_{n}\right)}_{j,v}{\left(\eta \right)}_{j\sigma ,\varrho }}{\prod_{u=1}^{k}\;{\left(\tau_{u}\right)}_{j,v}\Gamma_{\varrho }\left(\vartheta j+\varsigma +\varrho \right)}.\frac{z^{j}}{j!}+\sum_{j=0}^{\infty }\;\;\frac{\vartheta j\prod_{n=1}^{l}\;{\left(\varpi_{n}\right)}_{j,v}{\left(\eta \right)}_{j\sigma ,\varrho }}{\prod_{u=1}^{k}\;{\left(\tau_{u}\right)}_{j,v}\Gamma_{\varrho }\left(\vartheta j+\varsigma +\varrho \right)}.\frac{z^{j}}{j!}$$

$$\;=\sum_{j=0}^{\infty }\;\;\frac{\left(\vartheta j+\varsigma \right)\prod_{n=1}^{l}\;{\left(\varpi_{n}\right)}_{j,v}{\left(\eta \right)}_{j\sigma ,\varrho }}{\prod_{u=1}^{k}\;{\left(\tau_{u}\right)}_{j,v}\Gamma_{\varrho }\left(\vartheta j+\varsigma +\varrho \right)}.\frac{z^{j}}{j!}$$

$$\;=\sum_{j=0}^{\infty }\;\;\frac{\left(\vartheta j+\varsigma \right)\prod_{n=1}^{l}\;{\left(\varpi_{n}\right)}_{j,v}{\left(\eta \right)}_{j\sigma ,\varrho }}{\left(\vartheta j+\varsigma \right)\prod_{u=1}^{k}\;{\left(\tau_{u}\right)}_{j,v}\Gamma_{\varrho }\left(\vartheta j+\varsigma \right)}.\frac{z^{j}}{j!}=SM_{\tau ,\varsigma ,\vartheta }^{\varpi ,\eta }\left(j,\sigma ,v,\varrho ;z\right).$$

Theorem 2 *Let* η,σ,ϑ,ς∈C,min{R(ϑ) *,* R(ς),R(η)}>0 *and* ϱ∈R, *then*

(18) $${\left(\frac{d}{dz}\right)}^{m}SM_{\tau ,\varsigma ,\vartheta }^{\varpi ,\eta }\left(j,\sigma ,v,\varrho ;z\right)=\frac{\prod_{n=1}^{l}\;{\left(\varpi_{n}\right)}_{m,v}{\left(\eta \right)}_{k\sigma ,\varrho }}{\prod_{u=1}^{k}\;{\left(\tau_{u}\right)}_{m,v}}SM_{\tau +mv,\theta m+\varsigma ,\vartheta }^{\varpi +mv,\eta +m\varrho }\left(j,\sigma ,v,\varrho ;z\right).$$

*Proof.*

$${\left(\frac{d}{dz}\right)}^{m}SM_{\tau ,\varsigma ,\vartheta }^{\varpi ,\eta }\left(j,\sigma ,v,\varrho ;z\right)={\left(\frac{d}{dz}\right)}^{m}\sum_{j=0}^{\infty }\;\;\frac{\prod_{n=1}^{l}\;{\left(\varpi_{n}\right)}_{j,v}{\left(\eta \right)}_{j\sigma ,\varrho }}{\prod_{u=1}^{k}\;{\left(\tau_{u}\right)}_{j,v}\Gamma_{\varrho }\left(\vartheta j+\varsigma \right)}.\frac{z^{j}}{j!}$$

$$\;=\sum_{j=m}^{\infty }\;\;\frac{\prod_{n=1}^{l}\;{\left(\varpi_{n}\right)}_{j,v}{\left(\eta \right)}_{j\sigma ,\varrho }}{\prod_{u=1}^{k}\;{\left(\tau_{u}\right)}_{j,v}\Gamma_{\varrho }\left(\vartheta j+\varsigma \right)}.\frac{j!z^{j-m}}{j!\left(j-m\right)!}=\sum_{j=0}^{\infty }\;\;\frac{\prod_{n=1}^{l}\;{\left(\varpi_{n}\right)}_{j+m,v}{\left(\eta \right)}_{\left(j+m\right)\sigma ,\varrho }}{\prod_{u=1}^{k}\;{\left(\tau_{u}\right)}_{j+m,v}\Gamma_{\varrho }\left(\vartheta \left(j+m\right)+\varsigma \right)}.\frac{z^{j}}{j!}$$

$$\;={\left(\eta \right)}_{m\sigma ,\varrho }\sum_{j=0}^{\infty }\;\;\frac{\prod_{n=1}^{l}\;{\left(\varpi_{n}\right)}_{j+m,v}{\left(\eta +m\varrho \right)}_{j\sigma ,\varrho }}{\prod_{u=1}^{k}\;{\left(\tau_{u}\right)}_{j+m,v}\Gamma_{\varrho }\left(\vartheta j+\vartheta m+\varsigma \right)}.\frac{z^{j}}{j!}.$$

By the properties ${\left(\gamma \right)}_{j+m,\varrho }={\left(\gamma \right)}_{m,\varrho }{\left(\gamma +m\varrho \right)}_{j,\varrho }$ and (7), then

$$\;=\frac{\prod_{n=1}^{l}\;{\left(\varpi_{n}\right)}_{m,v}{\left(\eta \right)}_{k\sigma ,\varrho }}{\prod_{u=1}^{k}\;{\left(\tau_{u}\right)}_{m,v}}\sum_{j=0}^{\infty }\;\;\frac{\prod_{n=1}^{l}\;{\left({\left(\varpi +mv\right)}_{n}\right)}_{j,v}{\left(\eta +m\varrho \right)}_{j\sigma ,\varrho }}{\prod_{u=1}^{k}\;{\left({\left(\tau +mv\right)}_{u}\right)}_{j,v}\Gamma_{\varrho }\left(\vartheta j+\vartheta m+\varsigma \right)}.\frac{z^{j}}{j!}$$

$$\;=\frac{\prod_{n=1}^{l}\;{\left(\varpi_{n}\right)}_{m,v}{\left(\eta \right)}_{k\sigma ,\varrho }}{\prod_{u=1}^{k}\;{\left(\tau_{u}\right)}_{m,v}}SM_{\tau +mv,\theta m+\varsigma ,\vartheta }^{\varpi +mv,\eta +m\varrho }\left(j,\sigma ,v,\varrho ;z\right).$$

Theorem 3 *Let* η,σ,ϑ,ς∈C,min{R(ϑ) *,* R(ς),R(η)}>0 *and* ϱ∈R, *then*

(19) $$SM_{\tau ,\varsigma ,\vartheta }^{\varpi ,\eta }\left(j,\sigma ,v,\varrho ;z\right)-SM_{\tau ,\varsigma ,\vartheta }^{\varpi ,\eta -\varrho }\left(j,\sigma ,v,\varrho ;z\right)=\frac{\prod_{n=1}^{l}\;{\left(\varpi_{n}\right)}_{v}}{\prod_{u=1}^{k}\;{\left(\tau_{u}\right)}_{v}}\sigma \varrho^{\sigma }z{\left(\frac{\eta }{\varrho }\right)}_{\sigma -1}SM_{\tau +v,\vartheta +\varsigma ,\vartheta }^{\varpi +v,\eta +\varrho \sigma -\sigma }\left(j,\sigma ,v,\varrho ;z\right).$$

*Proof.* By taking the right side for Eq. (19). Using the relation between the property (14) and $j\sigma {\left(x\right)}_{j\sigma -1}={\left(x\right)}_{j\sigma }-{\left(x-1\right)}_{j\sigma }$, we have to prove

$$\overset{ˇ}{Q}=\varrho^{1-\frac{\varsigma }{\varrho }}\left[SM_{\tau ,\frac{\varsigma }{\varrho },\frac{\vartheta }{\varrho }}^{\varpi ,\frac{\eta }{\varrho }}\left(j,\sigma ,v,\varrho ;\varrho^{\sigma -\frac{\vartheta }{\varrho }}z\right)-SM_{\tau ,\frac{\varsigma }{\varrho },\frac{\vartheta }{\varrho }}^{\varpi ,\frac{\eta -\varrho }{\varrho },}\left(j,\sigma ,v,\varrho ;\varrho^{\sigma -\frac{\vartheta }{\varrho }}z\right)\right]$$

$$=\varrho^{1-\frac{\varsigma }{\varrho }}\sum_{j=0}^{\infty }\;\;\frac{\prod_{n=1}^{l}\;{\left(\varpi_{n}\right)}_{j,v}{\left(\frac{\eta }{\varrho }\right)}_{j\sigma -1}{\left(\varrho^{\sigma -\frac{\vartheta }{\varrho }}z\right)}^{j}.j\sigma }{\prod_{u=1}^{k}\;{\left(\tau_{u}\right)}_{j,v}\Gamma_{\varrho }\left(\frac{\vartheta }{\varrho }j+\frac{\varsigma }{\varrho }\right)j!}=\sigma \varrho^{1-\frac{\varsigma }{\varrho }}\sum_{j=0}^{\infty }\;\;\frac{\prod_{n=1}^{l}\;{\left(\varpi_{n}\right)}_{j+1,v}{\left(\frac{\eta }{\varrho }\right)}_{\left(j+1\right)\sigma -1}{\left(\varrho^{\sigma -\frac{\vartheta }{\varrho }}z\right)}^{j}.\left(j+1\right)}{\prod_{u=1}^{k}\;{\left(\tau_{u}\right)}_{j+1,v}\Gamma_{\varrho }\left(\frac{\vartheta }{\varrho }j+\frac{\vartheta }{\varrho }+\frac{\varsigma }{\varrho }\right)\left(j+1\right)!}$$

$$=\sigma \varrho^{1+\sigma -\frac{\vartheta +\varsigma }{\varrho }}z\sum_{j=0}^{\infty }\;\;\frac{\prod_{n=1}^{l}\;{\left(\varpi_{n}\right)}_{j+1,v}{\left(\frac{\eta }{\varrho }\right)}_{j\sigma +\sigma -1}{\left(\varrho^{\sigma -\frac{\vartheta }{\varrho }}z\right)}^{j}}{\prod_{u=1}^{k}\;{\left(\tau_{u}\right)}_{j+1,v}\Gamma_{\varrho }\left(\frac{\vartheta }{\varrho }j+\frac{\vartheta +\varsigma }{\varrho }\right)\left(j\right)!}.$$

By properties ${\left(\alpha \right)}_{j+q}={\left(\alpha \right)}_{q}{\left(\alpha +q\right)}_{j}$ and ${\left(\gamma \right)}_{j+m,\varrho }={\left(\gamma \right)}_{m,\varrho }{\left(\gamma +m\varrho \right)}_{j,\varrho }$, we get

$$\overset{ˇ}{Q}=\frac{\prod_{n=1}^{l}\;{\left(\varpi_{n}\right)}_{v}}{\prod_{u=1}^{k}\;{\left(\tau_{u}\right)}_{v}}\sigma \varrho^{1+\sigma -\frac{\vartheta +\varsigma }{\varrho }}z{\left(\frac{\eta }{\varrho }\right)}_{\sigma -1}\sum_{j=0}^{\infty }\;\;\frac{\prod_{n=1}^{l}\;{\left({\left(\varpi +v\right)}_{n}\right)}_{j,v}{\left(\frac{\eta }{\varrho }+\sigma -1\right)}_{j\sigma }{\left(\varrho^{\sigma -\frac{\vartheta }{\varrho }}z\right)}^{j}}{\prod_{u=1}^{k}\;{\left({\left(\tau +v\right)}_{u}\right)}_{j,v}\Gamma_{\varrho }\left(\frac{\vartheta }{\varrho }j+\frac{\vartheta +\varsigma }{\varrho }\right)\left(j\right)!}.$$

Hence

$$\overset{ˇ}{Q}=\frac{\prod_{n=1}^{l}\;{\left(\varpi_{n}\right)}_{v}}{\prod_{u=1}^{k}\;{\left(\tau_{u}\right)}_{v}}\sigma \varrho^{1+\sigma -\frac{\vartheta +\varsigma }{\varrho }}z{\left(\frac{\eta }{\varrho }\right)}_{\sigma -1}SM_{\rho +v,\frac{\vartheta +\varsigma }{\varrho },\frac{\vartheta }{\varrho }}^{\varpi +v,\frac{\eta }{\varrho }-\varrho -1}\left(j,\sigma ,v,\varrho ;\varrho^{\sigma -\frac{\vartheta }{\varrho }}z\right).$$

By property (15), it follows that

$$\overset{ˇ}{Q}=\frac{\prod_{n=1}^{l}\;{\left(\varpi_{n}\right)}_{v}}{\prod_{u=1}^{k}\;{\left(\tau_{u}\right)}_{v}}\sigma \varrho^{\sigma }z{\left(\frac{\eta }{\varrho }\right)}_{\sigma -1}SM_{\tau +v,\vartheta +\varsigma ,\vartheta }^{\varpi +v,\eta +\varrho \sigma -\sigma }\left(j,\sigma ,v,\varrho ;z\right).$$

In the subsequent part, we exmine fractional integral operators in terms of SM-function.

## The SM-function with fractional integral operators

Theorem 4 *Let* η,σ,ϑ,ς∈C,min{R(ϑ) *,* R(ς),R(η)}>0 *and* ϱ∈R, *then*

(20) $$\int_{0}^{\infty }\;\;e^{-\frac{u}{z}}u^{\mu }SM_{\tau ,\varsigma ,\vartheta }^{\varpi ,\eta }\left(j,\sigma ,v,\varrho ;u\right)du=\frac{z^{\mu +1}\Gamma \left(\mu +1\right)}{{\left(\mu +1\right)}_{\varrho }}SM_{\tau ,\varsigma ,\vartheta }^{\varpi ,\eta }\left(j,\sigma ,v,\varrho ;z\right).$$

*Proof.* Consider the integral

$$\begin{array}{ccc} \int_{0}^{\infty }\;e^{-\frac{u}{z}}u^{\mu }SM_{\tau ,\varsigma ,\vartheta }^{\varpi ,\eta }\left(j,\sigma ,v,\varrho ;u\right)du & = & \int_{0}^{\infty }\;\;e^{-\frac{u}{z}}u^{\mu }\sum_{j=0}^{\infty }\;\;\frac{\prod_{n=1}^{l}\;{\left(\varpi_{n}\right)}_{j,v}{\left(\eta \right)}_{j\sigma ,\varrho }}{\prod_{u=1}^{k}\;{\left(\tau_{u}\right)}_{j,v}\Gamma_{\varrho }\left(\vartheta j+\varsigma \right)}.\frac{u^{j}}{j!}du\\ & = & \sum_{j=0}^{\infty }\;\;\frac{\prod_{n=1}^{l}\;{\left(\varpi_{n}\right)}_{j,v}{\left(\eta \right)}_{j\sigma ,\varrho }}{\prod_{u=1}^{k}\;{\left(\tau_{u}\right)}_{j,v}\Gamma_{\varrho }\left(\vartheta j+\varsigma \right)j!}\int_{0}^{\infty }\;\;e^{-\frac{u}{z}}u^{\mu +j}du. \end{array}$$

Let $\frac{u}{z}=\chi ,$ we have

$$A=\sum_{j=0}^{\infty }\;\;\frac{\prod_{n=1}^{l}\;{\left(\varpi_{n}\right)}_{j,v}{\left(\eta \right)}_{j\sigma ,\varrho }z^{\mu +j+1}}{\prod_{u=1}^{k}\;{\left(\tau_{u}\right)}_{j,v}\Gamma_{\varrho }\left(\vartheta j+\varsigma \right)j!}\int_{0}^{\infty }\;\;e^{-\chi }{\left(\chi \right)}^{\mu +j}d\chi .$$

By the ϱ-Gamma function

$$A=\frac{z^{\mu +1}\Gamma \left(\mu +1\right)}{{\left(\mu +1\right)}_{\varrho }}\sum_{j=0}^{\infty }\;\;\frac{\prod_{n=1}^{l}\;{\left(\varpi_{n}\right)}_{j,v}{\left(\eta \right)}_{j\sigma ,\varrho }z^{j}}{\prod_{u=1}^{k}\;{\left(\tau_{u}\right)}_{j,v}\Gamma_{\varrho }\left(\vartheta j+\varsigma \right)j!}=\frac{z^{\mu +1}\Gamma \left(\mu +1\right)}{{\left(\mu +1\right)}_{\varrho }}SM_{\tau ,\varsigma ,\vartheta }^{\varpi ,\eta }\left(j,\sigma ,v,\varrho ;z\right).$$

Theorem 5 *Let* η,σ,ϑ,ς∈C,min{R(ϑ) *,* R(ς),R(η)}>0,ξ,β∈R *with* ξ>β>0 *and* ϱ∈R, *then*

(21) $$\int_{0}^{1}\;\;{\left(1-u^{\frac{1}{\beta }}\right)}^{\xi -\beta -1}SM_{\tau ,\varsigma ,\vartheta }^{\varpi ,\eta }\left(j,\sigma ,v,\varrho ;zu^{\frac{1}{\beta }}\right)du=\beta B\left(\beta ,\xi -\beta \right)SM_{\tau ,\varsigma ,\xi ,\vartheta }^{\varpi ,\eta ,\beta }\left(j,\sigma ,v,\varrho ;z\right).$$

*Proof.* Consider

$$\begin{array}{ccc} Q & = & \int_{0}^{1}\;\;{\left(1-u^{\frac{1}{\beta }}\right)}^{\xi -\beta -1}SM_{\tau ,\varsigma ,\vartheta }^{\varpi ,\eta }\left(j,\sigma ,v,\varrho ;zu^{\frac{1}{\beta }}\right)du\\ & = & \int_{0}^{1}\;\;{\left(1-u^{\frac{1}{\beta }}\right)}^{\xi -\beta -1}\sum_{j=0}^{\infty }\;\;\frac{\prod_{n=1}^{l}\;{\left(\varpi_{n}\right)}_{j,v}{\left(\eta \right)}_{j\sigma ,\varrho }}{\prod_{u=1}^{k}\;{\left(\tau_{u}\right)}_{j,v}\Gamma_{\varrho }\left(\vartheta j+\varsigma \right)}.\frac{{\left(zu^{\frac{1}{\beta }}\right)}^{j}}{j!}du \end{array}$$

$$=\sum_{j=0}^{\infty }\;\;\frac{\prod_{n=1}^{l}\;{\left(\varpi_{n}\right)}_{j,v}{\left(\eta \right)}_{j\sigma ,\varrho }z^{j}}{\prod_{u=1}^{k}\;{\left(\tau_{u}\right)}_{j,v}\Gamma_{\varrho }\left(\vartheta j+\varsigma \right)j!}\int_{0}^{1}\;\;{\left(1-u^{\frac{1}{\beta }}\right)}^{\xi -\beta -1}u^{j\frac{1}{\beta }}du.$$

Put $u^{\frac{1}{\beta }}=\chi ,$ then

$$=\sum_{j=0}^{\infty }\;\;\frac{\prod_{n=1}^{l}\;{\left(\varpi_{n}\right)}_{j,v}{\left(\eta \right)}_{j\sigma ,\varrho }z^{j}\beta }{\prod_{u=1}^{k}\;{\left(\tau_{u}\right)}_{j,v}\Gamma_{\varrho }\left(\vartheta j+\varsigma \right)j!}\int_{0}^{1}\;\;{\left(1-\chi \right)}^{\xi -\beta -1}\chi^{j+\beta -1}d\chi .$$

Using the beta function

$$Q=\frac{\beta \Gamma \left(\xi -\beta \right)\Gamma \left(\beta \right)}{\Gamma \left(\xi \right)}\sum_{j=0}^{\infty }\;\;\frac{\prod_{n=1}^{l}\;{\left(\varpi_{n}\right)}_{j,v}{\left(\eta \right)}_{j\sigma ,\varrho }z^{j}{\left(\beta \right)}_{j}}{\prod_{u=1}^{k}\;{\left(\tau_{u}\right)}_{j,v}\Gamma_{\varrho }\left(\vartheta j+\varsigma \right){\left(\xi \right)}_{j}j!}=\beta B\left(\beta ,\xi -\beta \right)SM_{\rho ,\varsigma ,\xi ,\vartheta }^{\varpi ,\eta ,\beta }\left(j,\sigma ,v,\varrho ;z\right).$$

Theorem 6 *Let* η,σ,ϑ,ς∈C,min{R(ϑ) *,* R(ς),R(η)}>0 *and* ϱ∈R, *then*

(22) $$\int_{0}^{\infty }\;\;e^{-uz}u^{\xi -1}SM_{\rho ,\varsigma ,\vartheta }^{\varpi ,\eta }\left(j,\sigma ,v,\varrho ;au\right)du=\frac{\Gamma \left(\xi \right)}{z^{\xi }}SM_{\tau ,\varsigma ,\vartheta }^{\varpi ,\xi ,\eta }\left(j,\sigma ,v,\varrho ;\frac{a}{z}\right).$$

*Proof.* Let

$$\begin{array}{ccc} Q_{1} & = & \int_{0}^{\infty }\;\;e^{-uz}u^{\xi -1}SM_{\tau ,\varsigma ,\vartheta }^{\varpi ,\eta }\left(j,\sigma ,v,\varrho ;au\right)du\\ & = & \int_{0}^{\infty }\;\;e^{-uz}u^{\xi -1}\sum_{j=0}^{\infty }\;\;\frac{\prod_{n=1}^{l}\;{\left(\varpi_{n}\right)}_{j,v}{\left(\eta \right)}_{j\sigma ,\varrho }}{\prod_{u=1}^{k}\;{\left(\tau_{u}\right)}_{j,v}\Gamma_{\varrho }\left(\vartheta j+\varsigma \right)}.\frac{{\left(au\right)}^{j}}{j!}du\\ & = & \sum_{j=0}^{\infty }\;\;\frac{\prod_{n=1}^{l}\;{\left(\varpi_{n}\right)}_{j,v}{\left(\eta \right)}_{j\sigma ,\varrho }a^{j}}{\prod_{u=1}^{k}\;{\left(\tau_{u}\right)}_{j,v}\Gamma_{\varrho }\left(\vartheta j+\varsigma \right)j!}\int_{0}^{\infty }\;\;e^{-uz}u^{\xi +j-1}du. \end{array}$$

Let uz=χ, then

$$\begin{array}{ccc} Q_{1} & = & \frac{1}{z}\sum_{j=0}^{\infty }\;\;\frac{\prod_{n=1}^{l}\;{\left(\varpi_{n}\right)}_{j,v}{\left(\eta \right)}_{j\sigma ,\varrho }a^{j}}{\prod_{u=1}^{k}\;{\left(\tau_{u}\right)}_{j,v}\Gamma_{\varrho }\left(\vartheta j+\varsigma \right)j!}\int_{0}^{\infty }\;\;e^{-\chi }{\left(\frac{\chi }{z}\right)}^{\xi +j-1}d\chi \\ & = & \frac{\Gamma \left(\xi \right)}{z^{\xi }}\sum_{j=0}^{\infty }\;\;\frac{\prod_{n=1}^{l}\;{\left(\varpi_{n}\right)}_{j,v}{\left(\eta \right)}_{j\sigma ,\varrho }{\left(\xi \right)}_{j}{\left(\frac{a}{z}\right)}^{j}}{\prod_{u=1}^{k}\;{\left(\tau_{u}\right)}_{j,v}\Gamma_{\varrho }\left(\vartheta j+\varsigma \right)j!}\int_{0}^{\infty }\;\;e^{-\chi }{\left(\frac{\chi }{z}\right)}^{\xi +j-1}d\chi \\ & = & \frac{\Gamma \left(\xi \right)}{z^{\xi }}SM_{\tau ,\varsigma ,\vartheta }^{\varpi ,\xi ,\eta }\left(j,\sigma ,v,\varrho ;\frac{a}{z}\right). \end{array}$$

Theorem 7 *Let* η,σ,ϑ,ς∈C,min{R(ϑ) *,* $R\left(\varsigma \right),\;R\left(\eta \right)\}>0,\;|x^{\vartheta }|<1$ *and* ϱ∈R, *then*

(23) $$\int_{0}^{\infty }\;\;e^{-u}{\left(xu\right)}^{\frac{\varsigma }{\varrho }-1}SM_{\tau ,\varsigma ,\vartheta }^{\varpi ,\eta }\left(j,\sigma ,v,\varrho ;{\left(xu\right)}^{\frac{\vartheta }{\varrho }}\right)du=x^{\frac{\varsigma }{\varrho }-1}SM_{\tau }^{\varpi ,\eta }\left(j,\sigma ,v,\varrho ;{\left(x\right)}^{\frac{\vartheta }{\varrho }}\right).$$

*Proof.* Let

$$\begin{array}{ccc} Q_{2} & = & \int_{0}^{\infty }\;\;e^{-u}{\left(xu\right)}^{\frac{\varsigma }{\varrho }-1}SM_{\tau ,\varsigma ,\vartheta }^{\varpi ,\eta }\left(j,\sigma ,v,\varrho ;{\left(xu\right)}^{\frac{\vartheta }{\varrho }}\right)du\\ & = & \int_{0}^{\infty }\;\;e^{-u}{\left(xu\right)}^{\frac{\varsigma }{\varrho }-1}\sum_{j=0}^{\infty }\;\;\frac{\prod_{n=1}^{l}\;{\left(\varpi_{n}\right)}_{j,v}{\left(\eta \right)}_{j\sigma ,\varrho }}{\prod_{u=1}^{k}\;{\left(\tau_{u}\right)}_{j,v}\Gamma_{\varrho }\left(\vartheta j+\varsigma \right)}.\frac{{\left({\left(xu\right)}^{\frac{\vartheta }{\varrho }}\right)}^{j}}{j!}du\\ & = & x^{\frac{\varsigma }{\varrho }-1}\sum_{j=0}^{\infty }\;\;\frac{\prod_{n=1}^{l}\;{\left(\varpi_{n}\right)}_{j,v}{\left(\eta \right)}_{j\sigma ,\varrho }x^{\frac{\vartheta }{\varrho }j}}{\prod_{u=1}^{k}\;{\left(\tau_{u}\right)}_{j,v}\Gamma_{\varrho }\left(\vartheta j+\varsigma \right)j!}\int_{0}^{\infty }\;\;e^{-u}{\left(u\right)}^{\frac{\vartheta }{\varrho }j+\frac{\varsigma }{\varrho }-1}du. \end{array}$$

By the definition of ϱ-Gamma function, we obtain

$$\begin{array}{ccc} x^{\frac{\varsigma }{\varrho }-1}\sum_{j=0}^{\infty }\;\;\frac{\prod_{n=1}^{l}\;{\left(\varpi_{n}\right)}_{j,v}{\left(\eta \right)}_{j\sigma ,\varrho }x^{\frac{\vartheta }{\varrho }j}}{\prod_{u=1}^{k}\;{\left(\tau_{u}\right)}_{j,v}\Gamma_{\varrho }\left(\vartheta j+\varsigma \right)j!}\Gamma_{\varrho }\left(\vartheta j+\varsigma \right) & = & x^{\frac{\varsigma }{\varrho }-1}\sum_{j=0}^{\infty }\;\;\frac{\prod_{n=1}^{l}\;{\left(\varpi_{n}\right)}_{j,v}{\left(\eta \right)}_{j\sigma ,\varrho }x^{\frac{\vartheta }{\varrho }j}}{\prod_{u=1}^{k}\;{\left(\tau_{u}\right)}_{j,v}j!}\\ & = & x^{\frac{\varsigma }{\varrho }-1}SM_{\tau }^{\varpi ,\eta }\left(j,\sigma ,v,\varrho ;{\left(x\right)}^{\frac{\vartheta }{\varrho }}\right). \end{array}$$

When η=1,implies that the generalized k-hypergeometric function

$$Q_{2}={x^{\frac{\varsigma }{\varrho }-1}}_{\;l}F_{k,j,v}\left[\varpi_{n};\tau_{u};x^{\frac{\vartheta }{\varrho }}\right]$$

is satisfied.

The ϱ-Weyl fractional operator is studied in the subsequent section. Numerous researchers will find it easier to solve integral and differential issues with the introduction of the extended ϱ-Weyl fractional integral and the examination of its characteristics and outcomes. These findings will be extremely helpful in resolving issues with fractional differential mask-based paper texture enhancement for medical imaging [21].

## Results of ϱ-Weyl fractional operator

This section discusses some analytic consequences of ϱ-Weyl fractional operator, which provided by [27], in terms of SM-function (also see [10,11,28] for further explain).

Theorem 8 *Let* η,σ,ϑ,ς∈C,min{R(ϑ) *,* R(ς),R(η),R(κ),R(μ)}>0 *and* ϱ∈R. *Then*

(24) $$\frac{1}{\Gamma_{\varrho }\left(\kappa \right)}\int_{0}^{1}\;\;\mu^{\frac{\varsigma }{\varrho }-1}{\left(1-\mu \right)}^{\frac{\kappa }{\varrho }-1}SM_{\tau ,\varsigma ,\vartheta }^{\varpi ,\eta }\left(j,\sigma ,v,\varrho ;z\mu^{\frac{\vartheta }{\varrho }}\right)d\mu =\varrho SM_{\tau ,\varsigma +\kappa ,\vartheta }^{\varpi ,\eta }\left(j,\sigma ,v,\varrho ;z\right).$$

*Proof.* We have the left side for Eq. (24) that

$$\frac{1}{\Gamma_{\varrho }\left(\kappa \right)}\int_{0}^{1}\;\;\mu^{\frac{\varsigma }{\varrho }-1}{\left(1-\mu \right)}^{\frac{\kappa }{\varrho }-1}SM_{\tau ,\varsigma ,\vartheta }^{\varpi ,\eta }\left(j,\sigma ,v,\varrho ;z\mu^{\frac{\vartheta }{\varrho }}\right)d\mu$$

$$\;=\frac{1}{\Gamma_{\varrho }\left(\kappa \right)}\sum_{j=0}^{\infty }\;\;\frac{\prod_{n=1}^{l}\;{\left(\varpi_{n}\right)}_{j,v}{\left(\eta \right)}_{j\sigma ,\varrho }z^{j}}{\prod_{u=1}^{k}\;{\left(\tau_{u}\right)}_{j,v}\Gamma_{\varrho }\left(\vartheta j+\varsigma \right)j!}\int_{0}^{1}\;\mu^{\frac{\varsigma }{\varrho }-1}{\left(1-\mu \right)}^{\frac{\kappa }{\varrho }-1}\;\mu^{\frac{\vartheta }{\varrho }j}d\mu .$$

By (4), we obtain

$$\sum_{j=0}^{\infty }\;\frac{\prod_{n=1}^{l}\;{\left(\varpi_{n}\right)}_{j,v}{\left(\eta \right)}_{j\sigma ,\varrho }z^{j}\varrho^{2-\left(\frac{\vartheta }{\varrho }+\frac{\varsigma }{\varrho }+\frac{\kappa }{\varrho }\right)}}{\Gamma \left(\frac{\kappa }{\varrho }\right)\prod_{u=1}^{k}\;{\left(\tau_{u}\right)}_{j,v}\Gamma \left(\frac{\vartheta }{\varrho }j+\frac{\varsigma }{\varrho }\right)j!}\int_{0}^{1}\;\mu^{\frac{\vartheta }{\varrho }j+\frac{\varsigma }{\varrho }-1}{\left(1-\mu \right)}^{\frac{\kappa }{\varrho }-1}\;d\mu$$

$$\;=\varrho \sum_{j=0}^{\infty }\;\frac{\prod_{n=1}^{l}\;{\left(\varpi_{n}\right)}_{j,v}{\left(\eta \right)}_{j\sigma ,\varrho }z^{j}\varrho^{2-\left(\frac{\vartheta }{\varrho }+\frac{\varsigma }{\varrho }+\frac{\kappa }{\varrho }\right)}}{\Gamma \left(\frac{\kappa }{\varrho }\right)\prod_{u=1}^{k}\;{\left(\tau_{u}\right)}_{j,v}\Gamma \left(\frac{\vartheta }{\varrho }j+\frac{\varsigma }{\varrho }\right)j!}B\left(\frac{\vartheta }{\varrho }j+\frac{\varsigma }{\varrho };\frac{\kappa }{\varrho }\right).$$

We get the following result by applying the well-known relationships between the ϱ-Gamma and ϱ-Beta functions:

$$\frac{1}{\Gamma_{\varrho }\left(\kappa \right)}\int_{0}^{1}\;\;\mu^{\frac{\varsigma }{\varrho }-1}{\left(1-\mu \right)}^{\frac{\kappa }{\varrho }-1}SM_{\tau ,\varsigma ,\vartheta }^{\varpi ,\eta }\left(j,\sigma ,v,\varrho ;z\mu^{\frac{\vartheta }{\varrho }}\right)d\mu =\varrho \sum_{j=0}^{\infty }\;\frac{\prod_{n=1}^{l}\;{\left(\varpi_{n}\right)}_{j,v}{\left(\eta \right)}_{j\sigma ,\varrho }z^{j}\varrho^{1-\left(\frac{\vartheta }{\varrho }+\frac{\varsigma }{\varrho }+\frac{\kappa }{\varrho }\right)}}{\prod_{u=1}^{k}\;{\left(\tau_{u}\right)}_{j,v}\Gamma \left(\frac{\vartheta }{\varrho }j+\frac{\varsigma }{\varrho }+\frac{\kappa }{\varrho }\right)j!}$$

$$\;=\varrho \sum_{j=0}^{\infty }\;\frac{\prod_{n=1}^{l}\;{\left(\varpi_{n}\right)}_{j,v}{\left(\eta \right)}_{j\sigma ,\varrho }z^{j}}{\prod_{u=1}^{k}\;{\left(\tau_{u}\right)}_{j,v}\Gamma_{\varrho }\left(\vartheta j+\varsigma +\kappa \right)j!}=\varrho SM_{\tau ,\varsigma +\kappa ,\vartheta }^{\varpi ,\eta }\left(j,\sigma ,v,\varrho ;z\right).$$

Theorem 9 *Let* η,σ,ϑ,ς∈C,min{R(ϑ) *,* R(ς),R(η),R(κ),R(ζ)}>0 *and* ϱ∈R. *Then*

(25) $$WF_{\varrho }^{\kappa }\left(SM_{\tau ,\varsigma ,\vartheta }^{\varpi ,\eta }\left(j,\sigma ,v,\varrho ;{\left(\zeta +a\right)}^{-\varsigma }\right)\right)=\frac{\Gamma_{\varrho }\left(\varsigma \varrho -\kappa \right){\left(a+z\right)}^{\frac{\kappa }{\varrho }}}{\Gamma_{\varrho }\left(\varsigma \varrho \right)}SM_{\tau ,\varsigma ,\vartheta }^{\varpi ,\eta }\left(j,\sigma ,v,\varrho ;{\left(a+z\right)}^{-\varsigma }\right).$$

*Proof.* We have the left side for Eq. (25) that

$$WF_{\varrho }^{\kappa }\left(SM_{\tau ,\varsigma ,\vartheta }^{\varpi ,\eta }\left(j,\sigma ,v,\varrho ;{\left(\zeta +a\right)}^{-\varsigma }\right)\right)$$

$$\;=\frac{1}{\varrho \Gamma_{\varrho }\left(\kappa \right)}\int_{z}^{\infty }\;{\left(\zeta -z\right)}^{\frac{\kappa }{\varrho }-1}\sum_{j=0}^{\infty }\;\frac{\prod_{n=1}^{l}\;{\left(\varpi_{n}\right)}_{j,v}{\left(\eta \right)}_{j\sigma ,\varrho }{\left(\zeta +a\right)}^{-\varsigma j}}{\prod_{u=1}^{k}\;{\left(\tau_{u}\right)}_{j,v}\Gamma_{\varrho }\left(\vartheta j+\varsigma \right)j!}d\zeta$$

$$\;=\frac{1}{\varrho \Gamma_{\varrho }\left(\kappa \right)}\sum_{j=0}^{\infty }\;\frac{\prod_{n=1}^{l}\;{\left(\varpi_{n}\right)}_{j,v}{\left(\eta \right)}_{j\sigma ,\varrho }}{\prod_{u=1}^{k}\;{\left(\tau_{u}\right)}_{j,v}\Gamma_{\varrho }\left(\vartheta j+\varsigma \right)j!}\int_{z}^{\infty }\;{\left(\zeta -z\right)}^{\frac{\kappa }{\varrho }-1}{\left(\zeta +a\right)}^{-\varsigma j}d\zeta .$$

Let $\chi =\frac{\zeta -z}{\zeta +a},$ we get $\zeta =\frac{z+a\chi }{1-\chi },\;d\zeta =\frac{a+z}{{\left(1-\chi \right)}^{2}}d\chi$ and

$$WF_{\varrho }^{\kappa }\left(SM_{\tau ,\varsigma ,\vartheta }^{\varpi ,\eta }\left(j,\sigma ,v,\varrho ;{\left(\zeta +a\right)}^{-\varsigma }\right)\right)$$

$$\;=\frac{1}{\Gamma_{\varrho }\left(\kappa \right)}\sum_{j=0}^{\infty }\;\frac{\prod_{n=1}^{l}\;{\left(\varpi_{n}\right)}_{j,v}{\left(\eta \right)}_{j\sigma ,\varrho }{\left(a+z\right)}^{\frac{\kappa }{\varrho }-\varsigma j}}{\prod_{u=1}^{k}\;{\left(\tau_{u}\right)}_{j,v}\Gamma_{\varrho }\left(\vartheta j+\varsigma \right)j!}\frac{1}{\varrho }\int_{0}^{1}\;\chi^{\frac{\kappa }{\varrho }-1}{\left(1-\chi \right)}^{\varsigma -\frac{\kappa }{\varrho }-1}d\chi$$

$$\;=\frac{1}{\Gamma_{\varrho }\left(\kappa \right)}\sum_{j=0}^{\infty }\;\frac{\prod_{n=1}^{l}\;{\left(\varpi_{n}\right)}_{j,v}{\left(\eta \right)}_{j\sigma ,\varrho }{\left(a+z\right)}^{\frac{\kappa }{\varrho }-\varsigma j}}{\prod_{u=1}^{k}\;{\left(\tau_{u}\right)}_{j,v}\Gamma_{\varrho }\left(\vartheta j+\varsigma \right)j!}B_{\varrho }\left(\kappa ,\varsigma \varrho -\kappa \right),$$

where $B_{\varrho }$ is the ϱ-Beta function.

$$WF_{\varrho }^{\kappa }\left(SM_{\tau ,\varsigma ,\vartheta }^{\varpi ,\eta }\left(j,\sigma ,v,\varrho ;{\left(\zeta +a\right)}^{-\varsigma }\right)\right)=\frac{1}{\Gamma_{\varrho }\left(\kappa \right)}\sum_{j=0}^{\infty }\;\frac{\prod_{n=1}^{l}\;{\left(\varpi_{n}\right)}_{j,v}{\left(\eta \right)}_{j\sigma ,\varrho }{\left(a+z\right)}^{\frac{\kappa }{\varrho }-\varsigma j}}{\prod_{u=1}^{k}\;{\left(\tau_{u}\right)}_{j,v}\Gamma_{\varrho }\left(\vartheta j+\varsigma \right)j!}B_{\varrho }\left(\kappa ,\delta \varrho -\kappa \right)$$

$$\;=\frac{1}{\Gamma_{\varrho }\left(\kappa \right)}\sum_{j=0}^{\infty }\;\frac{\prod_{n=1}^{l}\;{\left(\varpi_{n}\right)}_{j,v}{\left(\eta \right)}_{j\sigma ,\varrho }{\left(a+z\right)}^{\frac{\kappa }{\varrho }-\varsigma j}}{\prod_{u=1}^{k}\;{\left(\tau_{u}\right)}_{j,v}\Gamma_{\varrho }\left(\vartheta j+\varsigma \right)j!}\frac{\Gamma_{\varrho }\left(\kappa \right)\Gamma_{\varrho }\left(\varsigma \varrho -\kappa \right)}{\Gamma_{\varrho }\left(\varsigma \varrho \right)}=\sum_{j=0}^{\infty }\;\frac{\prod_{n=1}^{l}\;{\left(\varpi_{n}\right)}_{j,v}{\left(\eta \right)}_{j\sigma ,\varrho }{\left(a+z\right)}^{\frac{\kappa }{\varrho }-\varsigma j}}{\prod_{u=1}^{k}\;{\left(\rho_{u}\right)}_{j,v}\Gamma_{\varrho }\left(\vartheta j+\varsigma \right)j!}\frac{\Gamma_{\varrho }\left(\varsigma \varrho -\kappa \right)}{\Gamma_{\varrho }\left(\varsigma \varrho \right)}$$

$$\;=\frac{\Gamma_{\varrho }\left(\varsigma \varrho -\kappa \right){\left(a+z\right)}^{\frac{\kappa }{\varrho }}}{\Gamma_{\varrho }\left(\varsigma \varrho \right)}SM_{\tau ,\varsigma ,\vartheta }^{\varpi ,\eta }\left(j,\sigma ,v,\varrho ;{\left(a+z\right)}^{-\varsigma }\right).$$

Theorem 10 *Let* ϖ,η,τ,ϑ,ς∈C,min{R(ϑ) *,* R(ς),R(η),R(κ),R(ζ)}>0 *and* ϱ∈R, *then*

(26) $$WF_{\varrho }^{\kappa }\left(\zeta^{-\frac{\kappa }{\varrho }-\frac{\varsigma }{\varrho }}SM_{\tau ,\varsigma ,\vartheta }^{\varpi ,\eta }\left(j,\sigma ,v,\varrho ;u\zeta^{-\frac{\vartheta }{\varrho }}\right)\right)=z^{-\frac{\varsigma }{\varrho }}SM_{\tau ,\varsigma +\kappa ,\vartheta }^{\varpi ,\eta }\left(j,\sigma ,v,\varrho ;uz^{-\frac{\vartheta }{\varrho }}\right).$$

*Proof.* Consider

$$WF_{\varrho }^{\kappa }\left(\zeta^{-\frac{\kappa }{\varrho }-\frac{\varsigma }{\varrho }}SM_{\tau ,\varsigma ,\vartheta }^{\varpi ,\eta }\left(j,\sigma ,v,\varrho ;u\zeta^{-\frac{\vartheta }{\varrho }}\right)\right)=\frac{1}{\varrho \Gamma_{\varrho }\left(\kappa \right)}\int_{z}^{\infty }\;{\left(\zeta -z\right)}^{\frac{\kappa }{\varrho }-1}\zeta^{-\kappa -\frac{\varsigma }{\varrho }}\sum_{j=0}^{\infty }\;\frac{\prod_{n=1}^{l}\;{\left(\varpi_{n}\right)}_{j,v}{\left(\eta \right)}_{j\sigma ,\varrho }u^{j}\zeta^{-\frac{\vartheta }{\varrho }j}}{\prod_{u=1}^{k}\;{\left(\tau_{u}\right)}_{j,v}\Gamma_{\varrho }\left(\vartheta j+\varsigma \right)j!}d\zeta$$

$$\;=\frac{1}{\varrho \Gamma_{\varrho }\left(\kappa \right)}\sum_{j=0}^{\infty }\;\frac{\prod_{n=1}^{l}\;{\left(\varpi_{n}\right)}_{j,v}{\left(\eta \right)}_{j\sigma ,\varrho }u^{j}}{\prod_{u=1}^{k}\;{\left(\tau_{u}\right)}_{j,v}\Gamma_{\varrho }\left(\vartheta j+\varsigma \right)j!}\int_{z}^{\infty }\;{\left(\zeta -z\right)}^{\frac{\kappa }{\varrho }-1}\zeta^{-\kappa -\frac{\varsigma }{\varrho }-\frac{\vartheta }{\varrho }j}dv.$$

Put $\chi =\frac{\zeta -z}{\zeta }$, we obtain

$$WF_{\varrho }^{\kappa }\left(\zeta^{-\frac{\kappa }{\varrho }-\frac{\varsigma }{\varrho }}SM_{\tau ,\varsigma ,\vartheta }^{\varpi ,\eta }\left(j,\sigma ,v,\varrho ;u\zeta^{-\frac{\vartheta }{\varrho }}\right)\right)$$

$$\;=\frac{1}{\varrho \Gamma_{\varrho }\left(\kappa \right)}\sum_{j=0}^{\infty }\;\frac{\prod_{n=1}^{l}\;{\left(\varpi_{n}\right)}_{j,v}{\left(\eta \right)}_{j\sigma ,\varrho }u^{j}z^{-\frac{\varsigma }{\varrho }-\frac{\vartheta }{\varrho }j}}{\prod_{u=1}^{k}\;{\left(\tau_{u}\right)}_{j,v}\Gamma_{\varrho }\left(\vartheta j+\varsigma \right)j!}\int_{0}^{1}\;{\left(\chi \right)}^{\frac{\kappa }{\varrho }-1}{\left(1-\chi \right)}^{\frac{\vartheta }{\varrho }j-\frac{\varsigma }{\varrho }-1}d\chi$$

$$\;=\frac{1}{\varrho \Gamma_{\varrho }\left(\kappa \right)}\sum_{j=0}^{\infty }\;\frac{\prod_{n=1}^{l}\;{\left(\varpi_{n}\right)}_{j,v}{\left(\eta \right)}_{j\sigma ,\varrho }u^{j}z^{-\frac{\varsigma }{\varrho }-\frac{\vartheta }{\varrho }j}}{\prod_{u=1}^{k}\;{\left(\tau_{u}\right)}_{j,v}\Gamma_{\varrho }\left(\vartheta j+\varsigma \right)j!}B_{\varrho }\left(\kappa ,\vartheta j+\varsigma \right)$$

$$\;=z^{-\frac{\varsigma }{\varrho }}SM_{\tau ,\varsigma +\kappa ,\vartheta }^{\varpi ,\eta }\left(j,\sigma ,v,\varrho ;uz^{-\frac{\vartheta }{\varrho }}\right).$$

Theorem 11 *Let* ϖ,η,τ,ϑ,ς∈C,min{R(ϑ) *,* R(ς),R(η),R(κ),R(ζ)}>0 *and* ϱ∈R, *then*

(27) $$WF_{\varrho }^{\kappa }\left(SM_{\tau ,\varsigma ,\vartheta }^{\varpi ,\eta }\left(j,\sigma ,v,\varrho ;e^{-\omega z}\right)\right)=\frac{1}{\omega \varrho^{\frac{\kappa }{\varrho }}}SM_{\tau ,\varsigma ,\vartheta }^{\varpi ,\eta }\left(j,\sigma ,v,\varrho ;e^{-\omega z}\right).$$

*Proof. Consider*

$$W=WF_{\varrho }^{\kappa }\left(SM_{\tau ,\varsigma ,\vartheta }^{\varpi ,\eta }\left(j,\sigma ,v,\varrho ;e^{-\omega z}\right)\right)=\frac{1}{\varrho \Gamma_{\varrho }\left(\kappa \right)}\int_{z}^{\infty }\;{\left(\zeta -z\right)}^{\frac{\kappa }{\varrho }-1}\sum_{j=0}^{\infty }\;\frac{\prod_{n=1}^{l}\;{\left(\varpi_{n}\right)}_{j,v}{\left(\eta \right)}_{j\sigma ,\varrho }e^{-\omega \zeta j}}{\prod_{u=1}^{k}\;{\left(\tau_{u}\right)}_{j,v}\Gamma_{\varrho }\left(\vartheta j+\varsigma \right)j!}d\zeta$$

$$=\frac{1}{\varrho \Gamma_{\varrho }\left(\kappa \right)}\sum_{j=0}^{\infty }\;\frac{\prod_{n=1}^{l}\;{\left(\varpi_{n}\right)}_{j,v}{\left(\eta \right)}_{j\sigma ,\varrho }}{\prod_{u=1}^{k}\;{\left(\rho_{u}\right)}_{j,v}\Gamma_{\varrho }\left(\vartheta j+\varsigma \right)j!}\int_{z}^{\infty }\;{\left(\zeta -z\right)}^{\frac{\kappa }{\varrho }-1}e^{-\omega \zeta j}d\zeta .$$

Putting χ=ζ−z, we get

$$W=\frac{1}{\varrho \Gamma_{\varrho }\left(\kappa \right)}\sum_{j=0}^{\infty }\;\frac{\prod_{n=1}^{l}\;{\left(\varpi_{n}\right)}_{j,v}{\left(\eta \right)}_{j\sigma ,\varrho }}{\prod_{u=1}^{k}\;{\left(\tau_{u}\right)}_{j,v}\Gamma_{\varrho }\left(\vartheta j+\varsigma \right)j!}\int_{0}^{\infty }\;\chi^{\frac{\kappa }{\varrho }-1}e^{-\omega \left(\chi +z\right)j}d\chi .$$

Let ωχ=φ, therefore

$$W=\frac{1}{\varrho \Gamma_{\varrho }\left(\kappa \right)\omega^{\frac{\kappa }{\varrho }}}\sum_{j=0}^{\infty }\;\frac{\prod_{n=1}^{l}\;{\left(\varpi_{n}\right)}_{j,v}{\left(\eta \right)}_{j\sigma ,\varrho }e^{-\omega zj}}{\prod_{u=1}^{k}\;{\left(\tau_{u}\right)}_{j,v}\Gamma_{\varrho }\left(\vartheta j+\varsigma \right)j!}\int_{0}^{\infty }\;\varphi^{\frac{\kappa }{\varrho }-1}e^{-\varphi j}d\varphi .$$

From (4), we obtain

$$WF_{\varrho }^{\kappa }\left(SM_{\tau ,\varsigma ,\vartheta }^{\varpi ,\eta }\left(j,\sigma ,v,\varrho ;e^{-\omega z}\right)\right)=\frac{1}{\omega \varrho^{\frac{\kappa }{\varrho }}}SM_{\tau ,\varsigma ,\vartheta }^{\varpi ,\eta }\left(j,\sigma ,v,\varrho ;e^{-\omega z}\right).$$

## Conclusion

Within the extension of fractional integral and differential operators, we have established some novel findings of ϱ-fractional integral operators involving new extension of S-function in this paper. Furthermore, we discovered some unique instances of functions such as the M-series, R-function, and k-Mittag-Leffler function. The generalized k-Mittag-Leffler function findings given by [15] were obtained if we set n=u=σ=1,n=u=0,ϖ=γ,andτ=1. We reached good results about the ϱ-Weyl fractional operator and other fractional calculus operators. The investigated results are indicated by the generalized *k*-hypergeometric function, *k*-MLF and *R*-function. To demonstrate the probably enforcement of *SM*-type function, the investigations of fractional kinetic equations (FKEs) may be derived with the axuilary of Sumudu transform. The findings are also have considerable consequence as the solution of FKEs association variaty of other special functions. Moreover, numerous transforms such as Whittaker, Laplace and Fourier can be evaluated employing the *SM*-function.

## Limitations

Not applicable.

## Ethics authors statements

The platforms’ data redistribution policies were complied with.

## CRediT authorship contribution statement

**Sarem H. Hadi:** Conceptualization, Methodology, Writing – original draft, Visualization, Investigation, Software. **Khalid A. Challab:** Conceptualization, Methodology, Writing – original draft, Investigation, Software. **Ali Hasan Ali:** Conceptualization, Methodology, Writing – original draft, Visualization, Investigation, Software. **Abdullah A. Alatawi:** Conceptualization, Methodology, Writing – original draft, Investigation, Software.

## Declaration of competing interest

The authors declare that they have no known competing financial interests or personal relationships that could have appeared to influence the work reported in this paper.

## Data Availability

No data was used for the research described in the article.
